# Supplementary material for: SARS-CoV‑2 Spike Protein Amyloid Fibrils Impair Fibrin Formation and Fibrinolysis
Source: Biochemistry. 2025 Nov 26;64(24):4818–29. doi: 10.1021/acs.biochem.5c00550 (PMC12713721; doi:10.1021/acs.biochem.5c00550)
Supplement: Supplementary file 1 [file bi5c00550_si_001.docx]

***Supporting information***

SARS-CoV-2 spike protein amyloid fibrils impair fibrin formation and fibrinolysis

*Henrik Westman^1^, Per Hammarström^1,2^*, Sofie Nyström^1^**

*^1^Linköping University, IFM-Department of Physics, Chemistry, and Biology, Linköping University, 58183 Linköping, Sweden*

*^2^SciLifeLab, Linköping University, 58183 Linköping, Sweden*

**corresponding authors*

[*sofie.nystrom@liu.se*](mailto:sofie.nystrom@liu.se)

[*per.hammarstrom@liu.se*](mailto:per.hammarstrom@liu.se)

**Figure S1**

**
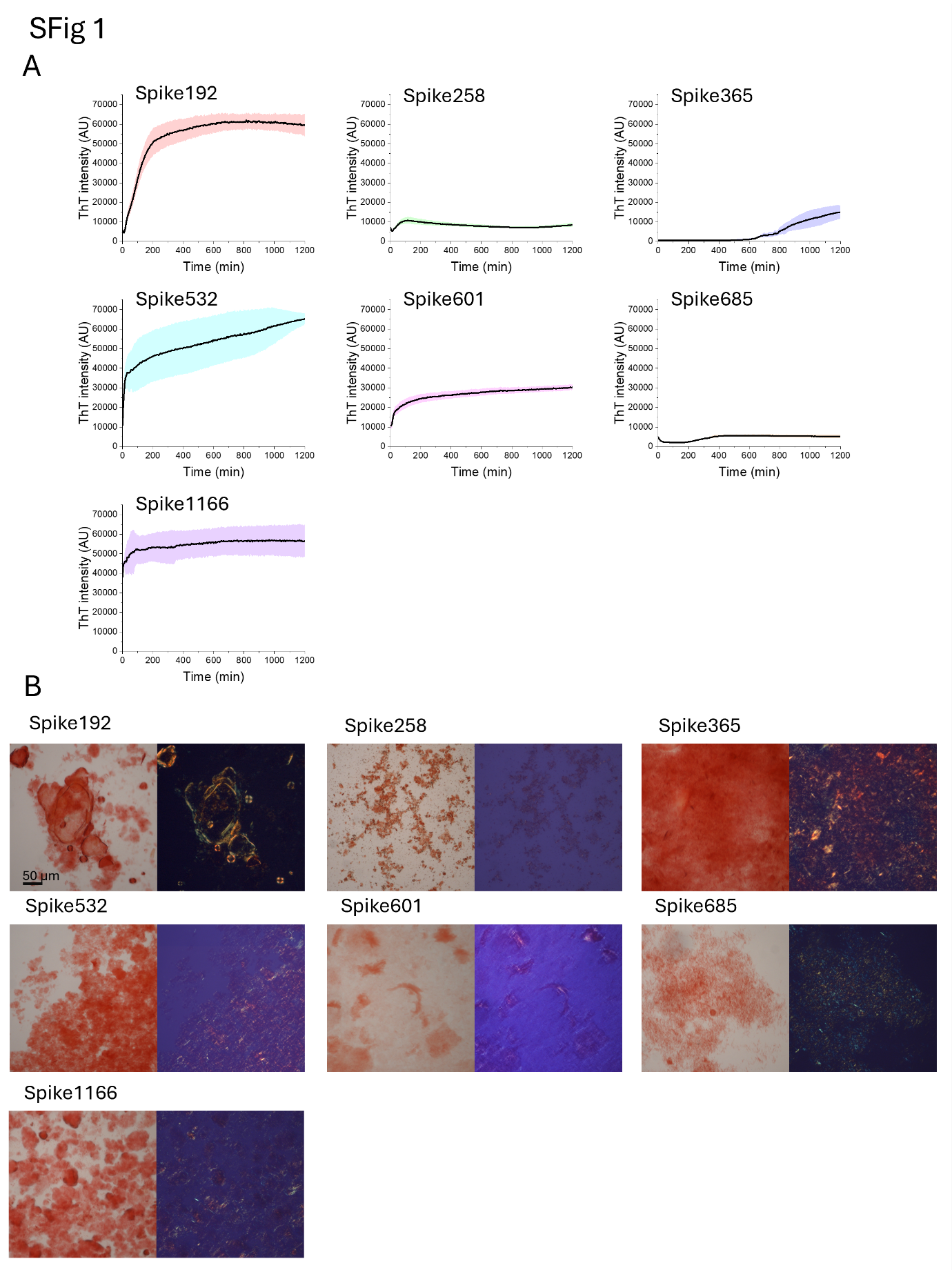
*Figure S1****A) Amyloid conversion of the seven Spike peptides was monitored by ThT intensity increase over time. B) Amyloid content at the endpoint of the Spike peptide fibrillation reaction was determined by light microscopy of Congo red stained samples without (left panel) and with (right panel) crossed polarizers. Fibrils were generated with spike peptide concentration of 0.5 mg/ml, in 5% HFIP in PBS buffer pH 7.5, 37 °C.*

**Figure S2**

***Figure S2*** *Spike amyloid fibrils were visible side by side with granular fibrin clots on the TEM grids of samples with lysed clots exemplified here by Spike685 amyloid fibrils to the left and granular fibrin clots to the right.*
